# How successful Bangladesh is in controlling the coronavirus pandemic?

**DOI:** 10.1186/s42269-020-00451-4

**Published:** 2020-11-23

**Authors:** Ayatullah Al Musabi Akanda, Redwan Ahmed

**Affiliations:** 1 Department of Economics, Government Ashek Mahmud College (Under Ministry of Education), Jamalpur, Bangladesh; 2 Department of Economics, Pabna University of Science and Technology, Pabna, Bangladesh

**Keywords:** COVID-19, SEIRD model, Simulation, Coronavirus, Pandemic

## Abstract

**Background:**

The reported number of COVID-19 patients increases on average along with the increased laboratory tests in Bangladesh implying a possibility of the spread of deadly coronavirus being out of control. Contrary to that, the government claims that it controls the spread of coronavirus through undertaking stringent policy measures. This different scenario leads this study on whether these measures have any positive impact on controlling the pandemic.

**Results:**

The results show that simulated number of patients (without policy measures) surpassed the actual number of patients (with policy measures) from the first week of July 2020 which may provide a signal about the positive impact of policy measures taken by the government.

**Conclusion:**

This study concludes that policy measures taken by the government are useful to some extent in controlling the coronavirus pandemic. As this pandemic lingers, people may lose their patience to stay at home. Consequently, some of the policies need further correction and change.

## Background

In December 2019, world experienced a new form of SARS-COV-2 virus in Wuhan city at the Hubei Province of China, which created COVID-19 disease (Li et al. 2020b). On January 30, 2020, WHO declared it as the Public Health Emergency of International Concern 'PHEIC' and subsequently as a pandemic on March 11, 2020 (Sohrabi et al. 2020). As of August 27, 2020, this virus spread over more than 213 countries and territories affecting 24,628,607 people, in which a total of 17,094,634 people gets recovered, 6,698,336 are still active cases, and death number rises to 835,637.^1^ This disease is highly infectious, and the growth rate of it follows the exponential curve, which is higher than the previously detected epidemic such as SARS and MERS (Peeri et al. 2020). Till today no effective and safe medicine is available, and all potential COVID-19 vaccines are under investigation (Campbell 2020). However, confinement is one of the best ways to slow it down (SCL Health 2020).

On March 07, 2020, this transmissible disease first detected in Bangladesh, the most densely populated country globally with more than one hundred sixty million in total living in an area of 147,570 km^2^ (Islam 2009). As a result, it creates a situation of a grievous threat for Bangladesh, where health facilities are not enough for the vast population. The density of the population, different transmission mechanisms depending on how far the virus could move through air and how long it could live in different surfaces, and the absence of vaccination make it challenging to deal with this disease (Cooper et al. 2020). Challenge in testing may cause infected people unnoticed (Salathé et al. 2020) and put others at risk (Roser et al. 2020), which could be minimized by enormous testing (Salathé et al. 2020). Combined with widespread testing and contact tracing, lockdown can subdue the pandemic (Giordano et al. 2020). Socioeconomic conditions may play a role of being tested and treated (Borjas 2020), and adequate support is required primarily to vulnerable communities to get the desired lockdown result.

### Measures taken by the country

Following the guidelines of WHO, Bangladesh adopted a holistic approach in formulating and implementing its own policy measures combining policy tools, such as regulation, economic, informational, educational, voluntary, and technological instruments to keep the pandemic at control. The government declared lockdown throughout the country starting from March 18, 2020, which subsequently extended until August 31, 2020.^2^ Thereafter, a list of virus hotspots was produced and marked as "red zones" for further lockdown based on the recommendations of the national technical committee formed to slow the spread of the virus down. To ensure stringent lockdown, the army deployed from March 24, 2020, and all forms of social, political, and religious gatherings have been forbidden. Banking, transportation, business activities, and shopping malls everything become stagnant. However, with the time being, the government ease activities step by step but not fully operational yet. The government also undertake a total of 19 different financial packages worth with Tk 1.04 trillion for homeless and low-income people to support them deal with challenging times during the pandemic.^3^ These packages include cash and food assistance, subsidized food items, financial aid, tax evasion, and other policy support to the business entities, ranges from small to large.

Raising awareness among mass people through providing relevant information is an important component of the policy measures taken by the government to deal with the pandemic. Required information has been provided through cell phone text messaging services (SMS), recorded voice services instead of usual caller tune, advertisement in print and electronic media, and leaflet distribution. As of August 20, 2020, a total of 215 government instructions and press releases were circulated to increase public consciousness. Awareness initiative also came up with an additional 37 guidelines and manual, and that addressed both psychological and pandemic inconveniences. Besides government agencies, many NGOs and voluntary groups distributed free hand sanitizer and face masks, and show how to use them for having protection from the infectious diseases.

During lockdown, online activities increased in Bangladesh. Lots of mobile applications have been launched, which are applicable in a different spectrum. Among these, 29 telemedicine, seven other mobile applications, two online helplines dedicated for foreign workers, 16 websites and portals, 10 COVID testing tools, and sevenchatbots, 245 digital transactions and shopping applications are still found operational on August 20, 2020.^4^ One of the salient featured applications is COVID-19 DSS (Digital Surveillance System), a passive (user must provide information) artificial intelligence system. It is useful to make awareness, digital screening, patient identification, and referral, identify risky zones, safety of health workers, and to amalgamate and analyze information. Corona tracker BD application is a risk assessment software that measures the probability of someone being infected.

### Overview of the country

As of August 27, 2020, Bangladesh conducted 9096 tests per million in 77 laboratories, where 43 is in the capital city of Dhaka, and 34 is outside Dhaka. Other South Asian countries like Sri Lanka tested 9962, India 27,912, Thailand 10,729, Nepal 21,744, Malaysia 36,742, Maldives 197,260 peoples per million.^5^ A total of 304,583 is known cases in which 193,458 (63.51%) have been recovered and 106,998 (35.13%) are still active cases with death number reaches to 4127 (1.35%). Table 1 represents biweekly recent scenario of infected as well as death cases in Bangladesh. According to the table, confirmed cases/laboratory test ratio increases overtime with an increased death rate. Regional spread of the disease is uneven in Bangladesh. According to the Directorate General of Health Services (DGHS) in Bangladesh, only Dhaka division accounts for nearly 69.5% of total COVID-19 patients whereas other administrative divisions such as Chattogram, Rajshahi, Khulna, Sylhet, Barisal-Rangpur together and Mymensingh division represent nearly 14.9%, 4.6%, 3.8%, 3.0%, 2.2% and 2.0%, respectively. Infected people are classified by gender to 71% males and 29% females, where the death rate for males share 79% of the total, which caused by behavioral and biological factors (Betron et al. 2020). However, 31–40 years aged group infected at 20.3% followed by 21–30 (14.4%), 41–50 (13.8%), 51–60 (10.8%) and 61–70 (5.7%), respectively. But most people died at the age group of 61–70 (23.7%), followed by 51–60 (20.3), 71–80 (15.1%), 41–50 (9.3%), 31–40 (4.6%) and more than 80 (4.3%) respectively.

**Table 1:** Summary statistics (mean value) of laboratory tests, confirmed cases and deaths. *Data Source*: Director-General of Health Service (Bangladesh) report (2020)

	Lab test (LT)	Confirmed	Death	Confirmed/LT	Death/LT
May 14–May 28	10,734 (2246.65)	1500.3 (342)	19.3 (4.13)	0.139	0.00179
May 29–June 13	15,699.42 (1006)	2840.78 (317.5)	37.78 (6.36)	0.181↑	0.00240↑
June 14–June 28	16,480.26 (1309)	3560.53 (303.5)	40 (5)	0.216↑	0.00242↑
June 29–July 13	14,850 (2449)	3274 (447)	43.53 (9.2)	0.221↑	0.00293↑

*Parentheses values represent Standard deviation

According to the DGHS, the overall attack rate (AR) is 1079.4 per million by 13.1% increase than the previous week (i.e., second week of August 2020). Highest AR observed in the Dhaka division (2916.5/million), which comprised of the contribution of Dhaka city (11,826.9), Narayanganj (1604.2), Faridpur (1475.4), Munshiganj (1405.9), and other cities with below 1,000 whereas the lowest reported in Tangail (226.4). Scenario of AR cases in other divisions are Chattogram (827.4), Sylhet (479), Barisal (421.7), Rajshahi (395.5), Khulna (386.6), Mymensingh (288.8), and the lowest in Rangpur (211). Although the growth factor (new cases on the previous day) is 1.16, the case doubling time in Bangladesh is nine days. In the Dhaka division, it is 8.3 days, nine days in Chattogram, 9.5–10 days in Khulna, Rajshahi, and Sylhet, and 10–11 days in Barisal, Rangpur, and Mymensingh. Bangladesh government set USD 2.36–5.89 and USD 47.15 as government and the private hospital charge, respectively, for the COVID-19 test fee from July 01, 2020. The DGHS report dated July 13, 2020 reported that 62,211 persons are in quarantine, and 334,044 people get released from the quarantine until the date. Besides, 16,230,490 phone calls received in four different numbers dedicated to provided telemedicine services by 4217 volunteers and 753,753 passengers screened in the international airports, seaports, land ports, and the railway station. Bangladesh kept 629 institutions standby for quarantine services, and 17,371 cases are in the institutional isolation now.

Bangladesh has taken a number of multidisciplinary measures which are primarily aligned with WHO guidelines to tackle the coronavirus pandemic. Despite having these stringent measures, number of confirmed patients increases on average along with the increased laboratory testing posing a question on whether these measures have any positive impact on controlling the pandemic. In this paper, our attempt is to examine how successful the measures taken till today are in dealing with the coronavirus pandemic. The paper is organized as follows: after introducing research methods which include data description, basic model, and parameter values section, result section is handled followed by a discussion, and a conclusion.

## Methods

### Data description

Our data collected from the Director-General of Health Service (Bangladesh) report and WHO situation report about Bangladesh.

### The basic model

Compartmental models (Kermack and McKendric 1927) are widely used to analyze current pandemics. We are using one of the versions of the SIR compartmental model known as the SEIRD model. Our model comes as follows:

1 $$S_{t} + E_{t} + I_{t} + R_{t} + D_{t} = N$$

Here, the variables stand for Susceptible, Exposed, Infectious, Recovered, Death, and total population. Our population moves from left-hand side variables, from one compartment to another compartment at a different rate based on several factors. The transition dynamics happened as follows:

At first, we assume all our peoples are susceptible, i.e., when *t* = 0, then $S_{t} = N$.

With the time being, peoples move from susceptible to exposed at the following way:

2 $${\text{d}}S_{t} {\text{/d}}t = - \beta_{t} SI{/}N$$

Here, beta is a time-varying parameter that depends on both policy, geography, and culture. This changed amount of the population goes to exposed states. After that, they move on to the next infectious stage.

3 $${\text{d}}E_{t} {\text{/d}}t = \beta_{t} SI{/}N - \sigma E$$

The exposed peoples move to the infectious group at the rate sigma. They are also subject to change at the rate gamma.

4 $${\text{d}}I_{t} {\text{/d}}t = \sigma E - \gamma I$$

These people then either recovered or died. The recovered peoples become:

5 $${\text{d}}R_{t} {\text{/d}}t = \gamma (1 - {\text{Death}})I$$

And, unfortunately, the number of dead people:

6 $${\text{d}}D_{t} {\text{/d}}t = ({\text{Death}})*\gamma *I$$

The parameters of transition, i.e., sigma, gamma, and death rate, are defined as biological characteristics. Movement from one to another compartment depends on these values. In our model, beta is affected by policy initiatives which subsequently affects all other transition parameters. We used this model for the simulation of the pandemic until the next year. This model is used by several other researchers (Atkeson 2020; Berger et al. 2020; Godio et al. 2020; Lin et al. 2020; López and Rodó 2020; Prem et al. 2020) in COVID-19 analysis.

### Parameter values

For the simulation, we set the value of the parameters. The reproduction rate (i.e., number of new infections generated by already infected persons) plays the most critical role in understanding the dynamics of the disease over time. It significantly varies from country to country. Kwok et al. (2020) studied 32 countries and found an effective reproduction rate for six countries is more than 4, in seventeen countries is between 2 and 4 and in nine countries is 1–2. Wang et al. (2020) estimated it as 3.1, 2.6, 1.9, and 0.9 or 0.5 at four different consecutive periods. However, the other researchers estimated the basic reproduction number at 2.2 (Li et al. 2020a), 2.28 (Zhang et al. 2020), 2.5 (Anderson et al. 2020), 2.68 (Wu et al. 2020), 3.8 (Read et al. 2020), and 6.47 (Tang et al. 2020). In the beginning, the reproduction rate was high, and after that, it decreases with time (Li et al. 2020b). Since the population density of Bangladesh (1265 per km^2^^6^) and Wuhan city of China (1152 per km^2^^7^) are close enough, our simulation took value of reproduction rate, 2.6, as same as the Wuhan city (Imai et al. 2020).

“The incubation period for COVID-19, which is the time between exposure to the virus (becoming infected) and symptom onset, is on average 5–6 days, however, it can be up to 14 days.”^8^ Moreover, Li et al. (2020a) argued incubation period for COVID-19 varies from 5 to 6 days. We set this parameter value (sigma) as 5.8 days, the average value also used by another researcher (Backer et al. 2020). Infectiousness sustains for 7–12 days for moderate and up to 2 weeks for extreme cases (Wölfel et al. 2020). The patient discharged from Wuhan hospital was 12.39 ± 4.77 days (Chen et al. 2020). Hence, set the value of our parameter (gamma) in this section as 12 days. The case-fatality ratio (i.e., death rate) varies from 4 to 10% (Chen et al. 2020; Yang et al. 2020). However, with different reproduction scenarios and pandemic situations, it varies. We are using 1.38% on an average death rate calculated based on China (Verity et al. 2020).

In our simulation, we assigned 267 people as susceptible, 39 infectious, five recovered, and four deaths. The government press release announced this figure on March 24, 2020.^9^ The reason behind choosing this date is, from that date, the government took intensive measures to curb the pandemic. As we simulate to get the long run likely outcome, our parameter value chosen to be the best fit in the long run rather than for a specific week.

## Result

**Fig. 1:**
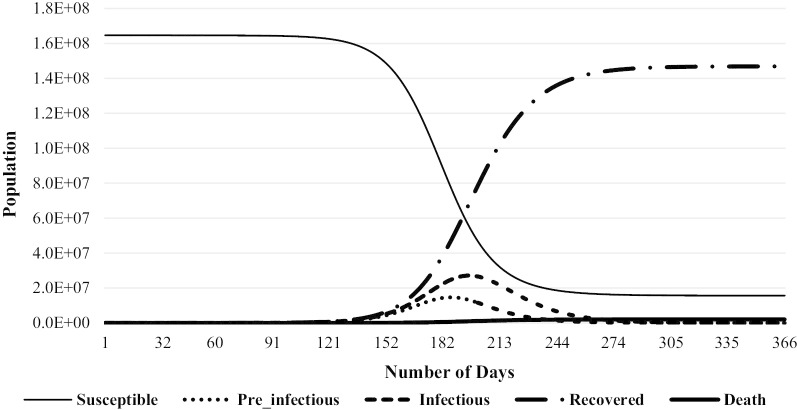
Simulated spread scenario of COVID-19

To understand the pandemic dynamics and the effectiveness of policy measures, we simulated the pandemic through the SEIRD model and compared it with the actual scenario. Figure 1 shows our simulated result. By the disease nature, peoples moved from one compartment to another compartment with a different rate. Figure 1 represents the disease simulation dynamics, with time being in the absence of any intervention and the policy implication.

The simulation dynamics show susceptible, pre-infectious, infectious, recovered, and dead people over a one-year period, from March 24, 2020, to March 23, 2021. Susceptible population started to fall drastically from the first week of August and continued till October. As this component decreases, the other components start to gain their values. Infectious people started to increase from August and reached to a peak in mid-October. The highest simulated death number was observed in September. After the month of November, curves become flatter.

As we got our values without government intervention, then we collate it with our available actual data. We compared the infected and dead peoples between two situations of simulated and actual cases. This comparison will postulate the effectiveness of our policy.

**Fig. 2:**
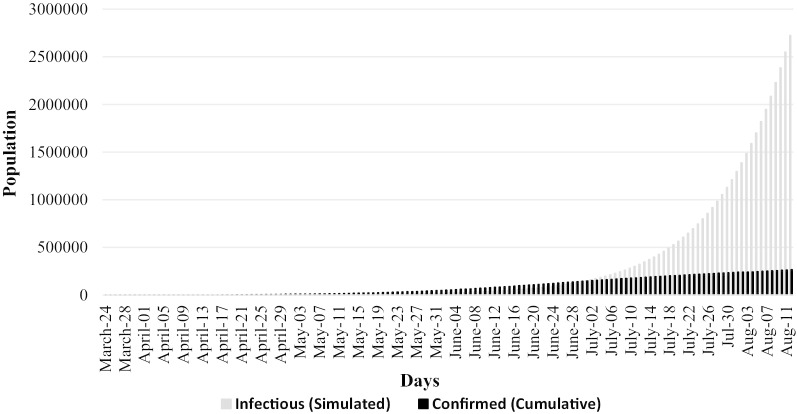
Simulated and actual number of patients

**Fig. 3:**
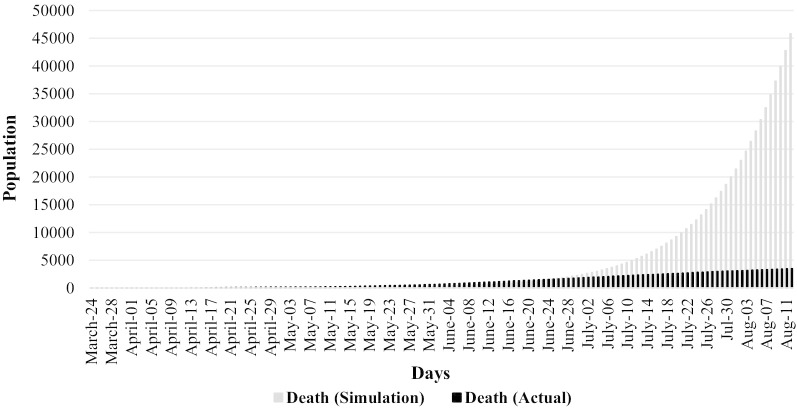
Simulated and actual cases of death

Figure 2 compares the simulated and actual number of COVID-19 patients until August 11, 2020. From July 02, 2020, total simulated patients surpassed confirmed patients, and with time being, this trend continued and confirmed the patient's figure got a flat curve scenario. Similar to Fig. 2, Fig. 3 depicts the simulated and actual number of deaths due to COVID-19 disease. Compared to simulated number of deaths, the actual number of dead people was higher from our very first day (i.e., March 24, 2020) to the last week of June. Afterwards, total simulated deaths surpassed the actual number of deaths which implies a positive outcome of government policy measures in controlling the coronavirus pandemic.

## Discussion

Our simulated values surpassed the actual values from the last week of June. Though actual number of infected and dead people still increase, daily numbers of these occurrences are stalled subsequently. As of July 02, 2020, in both cases of actual COVID-19 patients and actual deaths due to COVID-19 disease, our curves become flatter which implies that measures taken by the Bangladesh government are effective. Nearly six-month-long countrywide lockdown with army deployment in Bangladesh and further lockdown only on its "red zones" may have been an important reason behind the success in controlling the coronavirus pandemic. Already lockdown has been proven as an effective measure to subdue the spread of deadly coronavirus, and to reduce mortality notably (Papadopoulos et al. 2020). Nonetheless, it is not pragmatic to continue the measure, and make people stay at home for a longer period. People need movements to earn their livelihood, and to forget the severe discomfort they received during lockdown period. As of September 04, 2020, the google mobility index of Bangladesh shows, retail, and recreation come back to the baseline level, supermarket and pharmacy activities increase 18%, mobility in the parks increases 11%, presence in the workplaces is just 4% lower than the threshold.^10^ This threshold is defined by the median value of the five weeks from January 03, 2020 to February 06, 2020. Since pandemic situation is improving, the government may relax lockdown measure to boost up economic activities following the South Korean model in controlling the coronavirus pandemic. Universal health coverage, equitable access, and cost-effectiveness are the three pillars of the model (Jong-Wha 2020). According to Jong-Wha (2020), South Korea did “devise targeted strategies for effective healthcare delivery that go hand in hand with broader social- and economic-development efforts”.^11^ To sustain the success, the government needs testing, contact tracing and isolation, paid sick leave (Heymann et al. 2020), focus on underprivileged communities (Coetzee and Kagee 2020) and promoting health education. As people’s response to government policy in most cases is voluntary (Gupta et al. 2020) and its effectiveness depends on both policy and society (Weible et al. 2020). In our analysis, simulated values also show that it is not reached to the peak yet, and the nature of the diseases tells us in the absence of corrective measures, it may surge any time. Therefore, further pragmatic long-run strategies can keep the situation under control.

## Conclusion

Coronavirus pandemic becomes a global health issue affecting almost every nation of the world. Bangladesh located far away from Wuhan, the birthplace of the pandemic detected COVID-19 patient first time on March 07, 2020. Following that day, pandemic situation of the country started getting worse. The government of Bangladesh undertook several multidisciplinary policy measures to tackle the pandemic. In this article, we conducted a study to examine whether these measures taken were good enough to deal with the pandemic. After running a simulation of the COVID-19 based on the SEIRD model and comparing simulated values with actual values, we found government measures were useful to some extent. Controlling and mitigating the devastating effect of the pandemic, and providing supports to homeless and low-income people simultaneously is an arduous task for any government. The global crisis should be addressed globally through effective leadership, cooperation, and solidarity. Social distancing is the most useful way to curb the spread. Containment of the virus can be challenged through lockdown and effective lockdown preconditioned with available testing to check the pandemic. In some cases, only contact tracing and testing proven more effective than lockdown to keep the situation reasonable. In the long run, testing facilities of COVID-19 required to expand at the rural level to reduce total mortality by both public and private sectors. Hence, the government should reconsider the test fee and provide enormous test support to ordinary people irrespective of socioeconomic status and geographic location. As Bangladesh is about to relax almost every stringent measure, it should follow the success strategy of some other countries where lockdown is relatively less rigid.

## Data Availability

Not applicable.

## Abbreviations

COVID-19: Coronavirus disease of 2019
MERS: Middle East respiratory syndrome
NGO: Non-Governmental Organization
SARS: Severe acute respiratory syndrome
SEIRD: Susceptible-exposed-infected-recovered-dead
WHO: World Health Organization

## Footnotes

1 https://www.worldometers.info/coronavirus/.

2 https://cabinet.gov.bd/site/view/notices.

3 https://today.thefinancialexpress.com.bd/public/last-page/banks-fully-capable-of-executing-stimulus-packages-1596906916.

4 https://dghs.gov.bd/index.php/en/component/content/article?id=5393.

5 https://www.worldometers.info/coronavirus/.

6 https://www.worldometers.info/world-population/bangladesh-population/.

7 https://www.chinahighlights.com/wuhan/wuhan-facts.htm.

8 Situation Report – 73 by WHO; Dated 02 April 2020.

9 https://corona.gov.bd/press-release.

10 https://www.google.com/covid19/mobility/.

11 https://www.weforum.org/agenda/2020/08/south-korea-health-system-covid-19-coronavirus-pandemic/.
